# Prevalence of suicide attempts in individuals with schizophrenia: a meta-analysis of observational studies

**DOI:** 10.1017/S2045796019000313

**Published:** 2019-06-07

**Authors:** Li Lu, Min Dong, Ling Zhang, Xiao-Min Zhu, Gabor S. Ungvari, Chee H. Ng, Gang Wang, Yu-Tao Xiang

**Affiliations:** 1 Unit of Psychiatry, Institute of Translational Medicine, Faculty of Health Sciences, University of Macau, Macao SAR, China; 2 National Clinical Research Centre for Mental Disorders and Beijing Key Laboratory of Mental Disorders, Beijing Anding Hospital, and Advanced Innovation Centre for Human Brain Protection, Capital Medical University, Beijing, China; 3 Institute of Mental Health, Suzhou Psychiatric Hospital, The Affiliated Guangji Hospital of Soochow University, Jiangsu, China; 4 Division of Psychiatry, University of Western Australia Medical School, Perth, Australia & Graylands Hospital, Perth, Australia; 5 University of Notre Dame Australia/Marian Centre, Perth, Australia; 6 Department of Psychiatry, University of Melbourne, Melbourne, Victoria, Australia

**Keywords:** Meta-analysis, schizophrenia, suicide attempt

## Abstract

**Aims:**

Suicide attempt is an important indicator of suicide and potential future mortality. However, the prevalence of suicide attempts has been inconsistent across studies. This meta-analysis aimed to examine the prevalence of suicide attempts in individuals with schizophrenia and associated correlates.

**Methods:**

Relevant publications in Embase, PsycINFO, PubMed, Web of science and Cochrane were systematically searched. Data on the prevalence of suicide attempts in individuals with schizophrenia were pooled using a random-effects model.

**Results:**

Thirty-five studies with 16 747 individuals with schizophrenia were included. The pooled lifetime prevalence of suicide attempts was 26.8% (95% CI 22.1–31.9%; *I*^2^ = 97.0%), while the 1-year prevalence, 1-month prevalence and the prevalence of suicide attempts from illness onset were 3.0% (95% CI 2.3–3.7%; *I*^2^ = 95.6%), 2.7% (95% CI 2.1–3.4%; *I*^2^ = 78.5%) and 45.9% (95% CI 42.1–49.9%; *I*^2^ = 0), respectively. Earlier age of onset (*Q* = 4.38, *p* = 0.04), high-income countries (*Q* = 53.29, *p* < 0.001), North America and Europe and Central Asia (*Q* = 32.83, *p* < 0.001) were significantly associated with a higher prevalence of suicide attempts.

**Conclusions:**

Suicide attempts are common in individuals with schizophrenia, especially those with an early age of onset and living in high-income countries and regions. Regular screening and effective preventive measures should be implemented as part of the clinical care.

## Introduction

Schizophrenia is a chronic and severe psychiatric disorder with a massive global health burden, accounting for 7.4% (5.0–9.8) of disability-adjusted life years caused by mental and substance use disorders (Bhugra, 2005; Whiteford *et al*., 2013). Compared with the general population, persons with schizophrenia have 3.7 times higher risk of premature death (Olfson *et al*., 2015); men and women with schizophrenia have a reduced life-expectancy of around 19 and 16 years, respectively (Laursen, 2011). Among those with schizophrenia, the lifetime suicide rate is about 5% (Palmer *et al*., 2005; Hor and Taylor, 2010), and suicide is a major cause of premature death (Caldwell and Gottesman, 1992; Brown, 1997; Olfson *et al*., 2015). Prior suicide attempt is a major risk factor of suicide death (Hor and Taylor, 2010) and the lifetime prevalence of suicide attempts in individuals with schizophrenia ranged from 1.93% in Taiwan (Lee *et al*., 2012) to 55.1% in the USA (Roy *et al*., 1984).

Several demographic and clinical factors are associated with the risk of suicide attempts in persons with schizophrenia. For example, patients with comorbid depressive symptoms, a family history of suicide and multiple hospitalisations (Roy *et al*., 1984; Lee *et al*., 2012; Zhang *et al*., 2013) are at a higher risk of suicide attempts (Roy, 1983; Roy *et al*., 1984; Tremeau *et al*., 2005). Comorbid substance use (Togay *et al*., 2015; Fuller-Thomson and Hollister, 2016; Duko and Ayano, 2018) and more severe psychotic symptoms (Kao *et al*., 2012; Shrivastava *et al*., 2016) could also increase the risk of suicide attempts.

In order to develop effective preventive measures against suicide death, it is important to examine the epidemiology of suicide attempts in individuals with schizophrenia. However, the reported prevalence rates have been inconsistent across studies, probably due to discrepancy in study sampling, duration and regions with different economic levels. A meta-analysis of suicide-related behaviours in China found that the lifetime prevalence of suicide attempts was 14.6% in individuals with schizophrenia (Dong *et al*., 2017). To date there is no meta-analysis on the epidemiology of suicide attempts in person with schizophrenia worldwide. We thus conducted a meta-analysis of observational studies to examine the prevalence of suicide attempts in individuals with schizophrenia and associated factors.

## Methods

### Search strategy

This meta-analysis was performed in accordance with the Preferred Reporting Items for Systematic Reviews and Meta-Analyses (PRISMA) guidelines, and the protocol was registered in the International Prospective Register of Systematic Reviews (PROSPERO) with the registration number of CRD42018112863.

Two investigators (LL and MD) independently searched the databases of Embase, PsycINFO, PubMed, Web of science and Cochrane from their respective commencement date until 12 June 2018 using the following search terms: ((attempted suicide) OR (suicide attempt*)) AND (schizophrenia OR (schizophrenic disorder) OR (schizoaffective disorder) OR (Dementia Praecox)) AND (epidemiology OR (cross-sectional study) OR (cohort study) OR prevalence OR incidence OR rate).

### Study selection

Inclusion criteria were: (a) studies of individuals with a diagnosis of schizophrenia; (b) cross-sectional or cohort studies (only the baseline data of cohort studies were analysed); (c) studies reporting prevalence of suicide attempts or providing relevant data which enabled the calculation of prevalence of suicide attempts; (d) studies published in English. Secondary analyses of medical records alone or studies with very small sample size, no timeframe or special populations (such as twins and samples in veteran/military hospitals) were excluded. Studies with mixed samples were included if data on schizophrenia and related diagnoses (e.g. schizoaffective or schizophrenia spectrum disorders) were reported separately. In order to increase homogeneity, only data of schizophrenia were extracted for analyses.

In the initial search, the titles and abstracts of publications were independently screened, and then the full texts were read by two investigators (LL and MD) to identify eligible studies. If there were multiple publications based on the identical study sample, only the one with the most complete information was analysed. Any discrepancies in study search and selection were resolved by a discussion or a consultation with a senior investigator (YTX).

### Data extraction and quality assessment

Relevant data were independently extracted by the same two investigators (LL and MD), including country, study design, sample size and events of suicide attempts, mean age, gender proportion, source of patients (such as inpatients, outpatients, community or mixed), diagnostic criteria of schizophrenia, assessment tools and timeframe of suicide attempts. Study quality was also independently evaluated by the same two investigators using an eight-item instrument for quality assessment of epidemiological studies (Loney *et al*., 1998). The items are shown in online Supplementary Table S1. The total score ranged from 0 to 8.

### Statistical analysis

The prevalence and its 95% confidence intervals (CI) of suicide attempts were calculated using a random-effects model and Freeman Tukey double arcsine transformation (Freeman and Tukey, 1950). Heterogeneity between studies was measured by *τ*^2^ and *I*^2^ statistic, with *I*^2^> 50% indicating high heterogeneity (Higgins *et al*., 2003).

In order to explore the sources of heterogeneity, subgroup analyses and meta-regression analyses (at least ten studies are needed) were performed. Subgroup analyses were conducted for categorical variables, such as gender (female/male); source of patients (inpatients/outpatients/community/mixed); economic group (low income/lower middle income/upper middle income and high income) and region (sub-Saharan Africa/East Asia and Pacific/South Asia/Europe and Central Asia/North America) according to the classification of the World Bank; assessment tools of suicide attempt (interview or and records/others). As recommended previously (Higgins and Green, 2011), at least ten studies are needed to perform meta-regression analyses. The potential moderating effects of continuous variables on lifetime prevalence of suicide attempts, such as sample size, mean age, the proportion of female patients, publication year and assessment score were also examined in this meta-analysis.

Funnel plots and Egger's regression model (Egger *et al*., 1997) were used to test publication bias. Sensitivity analysis was implemented by removing each study sequentially to assess the consistency of the primary results. Comprehensive Meta-Analysis software version 2 (Biostat Inc., Englewood, New Jersey, USA) and STATA version 12.0 (Stata Corporation, College Station, Texas, USA) were used for analyses with the significance level as a *p* < 0.05 (two-tailed).

## Results

### Search results

**Fig. 1.**
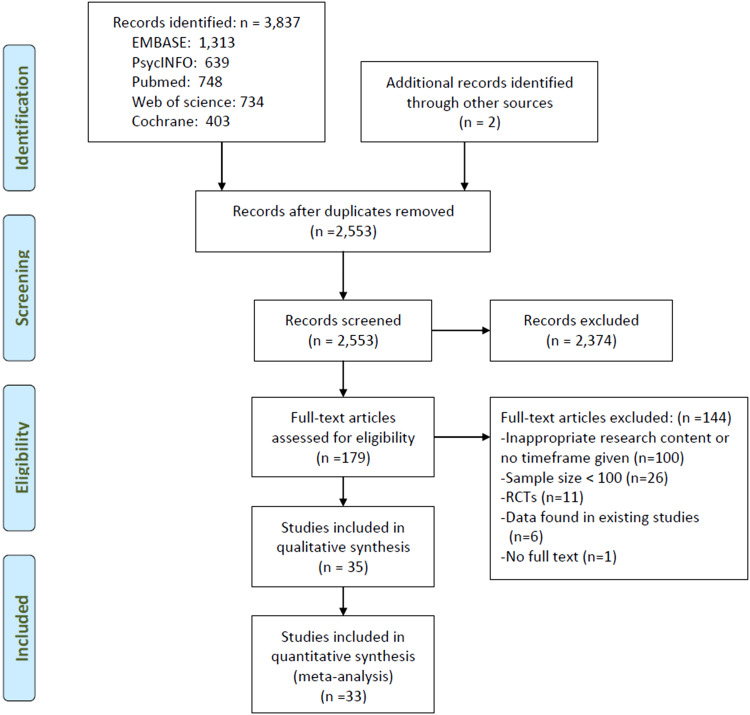
Flowchart of the selection of studies.

From a total of 3837 potential studies identified, 35 studies with 16 747 individuals with schizophrenia were included in the meta-analyses (Fig. 1). The full text of one study (Marcinko *et al*., 2008) could not be found and therefore was not included.

### Study characteristics and quality assessment

Study characteristics are shown in Table 1. The mean age was 40.1 years and women accounted for 37.1% of the whole sample. Twenty-eight studies (11 756 patients) reported the lifetime prevalence, one study reported both the lifetime and 1-month prevalence (Radomsky *et al*., 1999), two studies reported the 1-year prevalence (Tang *et al*., 2007; Lee *et al*., 2012) and one study reported the 1-month prevalence of suicide attempts (Malandain *et al*., 2018), and two studies reported the prevalence of suicide attempts since illness onset (Prasad and Kellner, 1988; Assefa *et al*., 2012). One study from India (Shrivastava *et al*., 2016) and another from Greece (Andriopoulos *et al*., 2011) reported the 6-month prevalence and the prevalence of suicide attempts during the prodromal period, respectively.

**Table 1.** Characteristics of the studies included in the meta-analysis

No.	First author (year)	References	Country^a^	Study design	Sample size	Age (years) M/R	Female (%)	Diagnostic criteria (SCH)^b^	Assessment tools (SA)^c^	Period experienced
1	Malandain 2018	(Malandain *et al*., 2018)	France	Cohort	1859	38.1	31.4	DSM-IV	Question	1 month
2	Duko 2018	(Duko and Ayano 2018)	Ethiopia	Cross-sectional	272	33.7	30.9	DSM-IV	CIDI	Lifetime
3	Jakhar 2017	(Jakhar *et al*., 2017)	India	Cross-sectional	270	NR	NR	DSM-IV	DIGS and records	Lifetime
4	Shrivastava 2016	(Shrivastava *et al*., 2016)	India	Cross-sectional	200	36.5	40.5	DSM-IV	Interview	6 months
5	Fuller-Thomson 2016	(Fuller-Thomson and Hollister, 2016)	Canada	Cross-sectional	101	NR	NR	Clinical diagnosis	Question	Lifetime
6	Fulginiti 2016	(Fulginiti and Brekke 2016)	USA	Cohort	166	33.6	24.1	SADS	SADS	Lifetime
7	Togay 2015	(Togay *et al*., 2015)	Turkey	Cohort	172	15–45	40.1	DSM-IV	Interview and records	Lifetime
8	Ran 2015	(Ran *et al*., 2015)	China	Cohort	510	⩾15	53.5	ICD-10	Interview	Lifetime
9	Finseth 2014	(Finseth *et al*., 2014)	Norway	Cross-sectional	338	NR	NR	DSM-IV	Interview	Lifetime
10	Zhang 2013	(Zhang *et al*., 2013)	China	Cross-sectional	520	49.4	33.5	DSM-IV	Interview and records	Lifetime
11	Tamminga 2013	(Tamminga *et al*., 2013)	USA	Case–control	361	NR	NR	DSM-IV	Interview	Lifetime
12	Polsinelli 2013	(Polsinelli *et al*., 2013)	Canada	Cross-sectional	234	37.0	29.5	DSM-IV	Interview	Lifetime
13	Ndetei 2013	(Ndetei *et al*., 2013)	Kenya	Cross-sectional	170	33.5	37.1	DSM-IV	Interview and records	Lifetime
14	Lee 2012	(Lee *et al*., 2012)	Taiwan, China	Cross-sectional	1655	43.9	40.9	Clinical diagnosis	Interview and records	1 year
15	Kao 2012	(Kao *et al*., 2012)	Taiwan, China	Cross-sectional	102	39.5	51.0	DSM-IV	Question	Lifetime
16	Assefa 2012	(Assefa *et al*., 2012)	Ethiopia	Cross-sectional	212	33.3	34.9	DSM-IV	Question	From on-set
17	Okusaga 2011	(Okusaga *et al*., 2011)	Germany	Cross-sectional	950	38.0	36.8	DSM-IV	Interview	Lifetime
18	Hung 2011	(Hung *et al*., 2011)	Taiwan, China	Cross-sectional	168	38.3	37.5	DSM-IV	Interview	Lifetime
19	Andriopoulos 2011	(Andriopoulos *et al*., 2011)	Greece	Case–control	106	29.6	30.2	DSM-IV	Interview	Prodromal phase
20	Uzun 2009	(Uzun *et al*., 2009)	Turkey	Cross-sectional	300	36.7	35.0	DSM-IV	Interview and records	Lifetime
21	Xiang 2008	(Xiang *et al*., 2008)	China	Cross-sectional	505	43.0	51.9	DSM-IV	Interview and records	Lifetime
22	Tang 2007	(Tang *et al*., 2007)	China	Cross-sectional	542	41.6	45.0	DSM-IV	Interview	1 year
23	Limosin 2007	(Limosin *et al*., 2007)	France	Cohort	3425	18–64	36.2	ICD-10	Interview	Lifetime
24	Tremeau 2005	(Tremeau *et al*., 2005)	France	Cross-sectional	160	34.2	27.5	SADS	Interview	Lifetime
25	De Luca 2005	(De Luca *et al*., 2005)	Canada	Cross-sectional	253	41.0	33.0	DSM	SCID-I	Lifetime
26	Ran 2004	(Ran *et al*., 2004)	China	Cross-sectional	145	32.2	49.0	DSM-IV	SAIS	Lifetime
27	Niehaus 2004	(Niehaus *et al*., 2004)	South Africa	Cross-sectional	454	NR	27.8	DIGS	DIGS	Lifetime
28	Kebede 2003	(Kebede *et al*., 2003)	Ethiopia	Cohort	312	NR	16.0	ICD-10/DSM-IV	Interview	Lifetime
29	Chong 2000	(Chong *et al*., 2000)	Singapore	Cross-sectional	338	47.1	36.1	DSM-IV	Interview	Lifetime
30	Radomsky 1999	(Radomsky *et al*., 1999)	USA	Cross-sectional	454	15–55	35.7	DSM-III	Interview and records	Lifetime and 1 month
31	Harkavy-Friedman 1999	(Harkavy-Friedman *et al*., 1999)	USA	Cross-sectional	112	NR	NR	DSM-III	DIGS	Lifetime
32	Dixon 1999	(Dixon *et al*., 1999)	USA	Cross-sectional	719	43.2	36.9	Clinical diagnosis	Question	Lifetime
33	Prasad 1988	(Prasad and Kellner, 1988)	UK	Cross-sectional	417	38.4	45.8	RDC	Interview	From on-set
34	Landmark 1987	(Landmark *et al*., 1987)	UK	Cross-sectional	118	38.4	61.0	12 systems	Interview	Lifetime
35	Roy 1984	(Roy *et al*., 1984)	USA	Cross-sectional	127	NR	42.5	RDC and DSM	Interview	Lifetime

M, mean; NR, not reported; R, range; SA, suicide attempt; SCH, schizophrenia.

a Country: UK, United Kingdom; USA, United States.

b Diagnostic criteria (SCH): DIGS, The Diagnostic Interview for Genetic Studies; DSM-III, Diagnostic and Statistical Manual of Mental Disorders, 3rd Edition; DSM-IV, Diagnostic and Statistical Manual of Mental Disorders, 4th edition; ICD-10, the 10th Revision of the International Statistical Classification of Diseases and Related Health. Problems; RDC, The Research Diagnostic Criteria; SADS, Schedule for Affective Disorders and Schizophrenia;12 systems, 12 systems for diagnosing schizophrenia.

c Assessment tools (SA): CIDI, The composite international diagnostic interview; DIGS, The Diagnostic Interview for Genetic Studies; SADS, Schedule for Affective. Disorders and Schizophrenia; SAIS, Suicide Attempts Investigation Schedule; SCID-I, The Structured Interview for Psychiatric Diagnosis.

Quality assessment of all the 35 studies ranged from 4 to 7; the details of quality assessment are shown in online Supplementary Table S1.

### Prevalence of suicide attempts

**Fig. 2.**
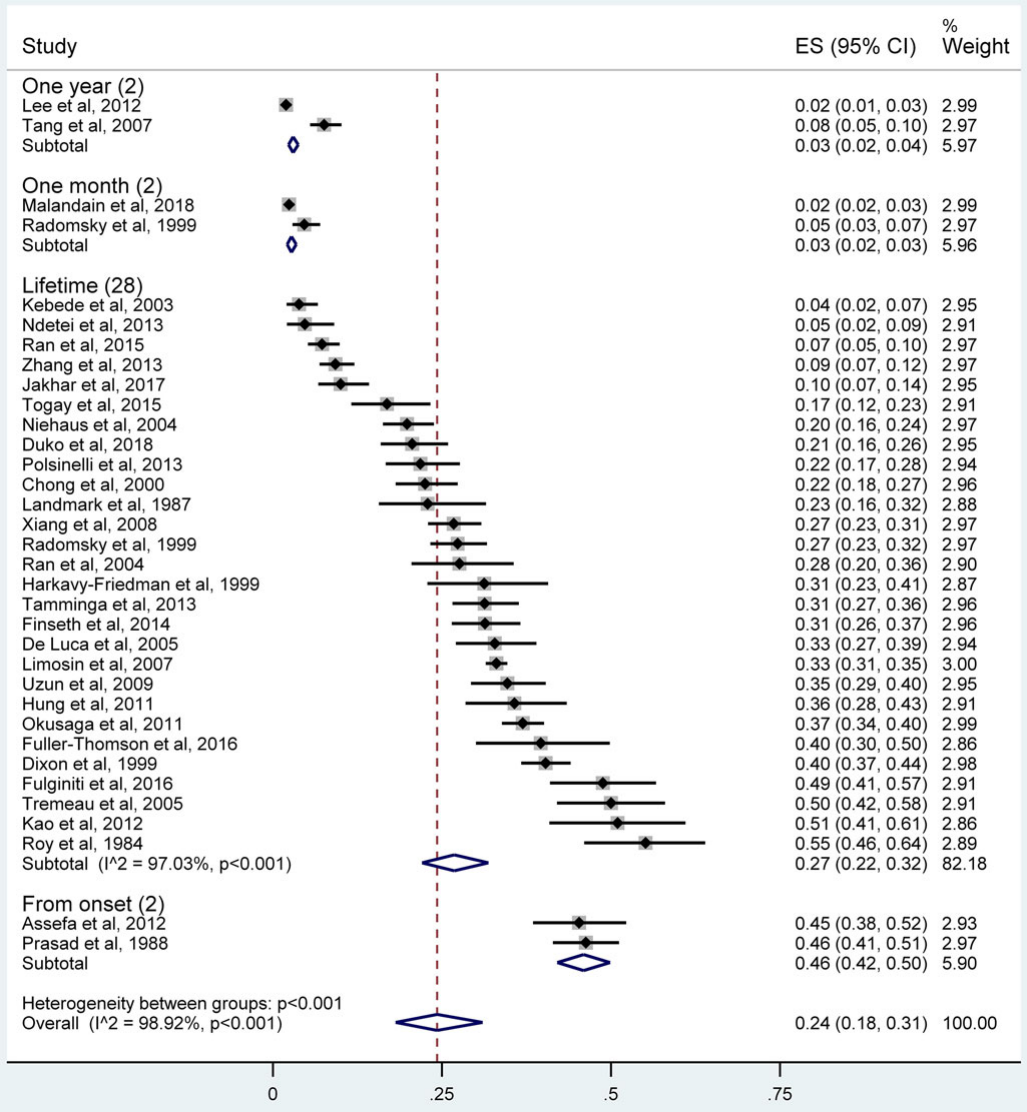
Forest plot of the prevalence of suicide attempts among individuals with schizophrenia.

The pooled lifetime prevalence of suicide attempts was 26.8% (95% CI 22.1–31.9%; *τ*^2^ = 0.019, *I*^2^ = 97.0%, *p* < 0.001), while the 1-year prevalence, 1-month prevalence and the prevalence of suicide attempts from illness onset in individuals with schizophrenia were 3.0% (95% CI 2.3–3.7%; *τ*^2^ = 0.002, *I*^2^ = 95.6%), 2.7% (95% CI 2.1–3.4%; *τ*^2^ = 0.0002, *I*^2^ = 78.5%) and 45.9% (95% CI 42.1–49.9%; *τ*^2^ = 0, *I*^2^ = 0), respectively (Fig. 2). The 6-month prevalence was 38% and the prevalence during the prodromal period was 7.5%.

### Subgroup and meta-regression analyses

The subgroup analyses of lifetime prevalence of suicide attempts are shown in Table 2. The prevalence in high-income countries (35.3%, 95% CI 31.7–38.9%) was significantly higher than those in lower economic level countries (*Q*  =  53.29, *p* < 0.001). Patients from North America (35.9%, 95% CI 29.8–42.2%) and Europe and Central Asia (32.2%, 95% CI 27.4–37.2%) were more likely to have suicide attempts than those from East Asia and Pacific (23.9%, 95% CI 14.3–35.2%), sub-Saharan Africa (11.0%, 95% CI 3.6–21.8%) and South Asia (10.0%, 95% CI 6.7–14.2%; *Q*  =  32.83, *p* < 0.001). Early onset of illness group (41.8%, 95% CI 30.7–53.5%) had more frequent suicide attempts than late-onset patients (23.6%, 95% CI 12.1–37.6%; *Q*  =  4.38, *p*  =  0.04).

**Table 2.** Subgroup analyses of the lifetime prevalence of suicide attempts in individuals with schizophrenia

Subgroups	Categories (number of studies)	Prevalence (%)	95% CI (%)		Events	Sample size	*τ*^2^ (*P*)	*I*^2^ (%, *P*)		*Q* (*p value between subgroups*)
Gender	Female (15)	28.2	21.0	36.1	928	3049	0.48 (0.489)	94.4	<0.001	1.03 (0.31)
	Male (15)	24.6	17.7	32.2	1400	5324		96.9	<0.001	
Mean age (years)	Age ⩽ 38.15 (8)	29.3	20.0	39.5	771	2397	0 (0.985)	96.1	<0.001	0.001 (0.97)
	Age > 38.15 (8)	29.1	19.9	39.4	771	2723		96.7	<0.001	
Source of patients	Inpatients (7)	25.9	14.2	39.6	446	1914		97.5	<0.001	1.85 (0.60)
	Outpatients (3)	27.3	10.5	48.4	214	877		97.5	<0.001	
	Community (4)	21.2	4.6	45.4	170	1089		98.5	<0.001	
	Mixed (4)	31.0	24.1	38.3	1618	4936		94.8	<0.001	
Duration of illness (years)	⩽15.8 (5)	33.7	26.4	41.4	627	1795	0.99 (0.804)	89.5	<0.001	0.69 (0.41)
	>15.8 (4)	27.8	12.5	46.5	236	1128		97.4	<0.001	
Age of onset (years)	⩽24.38 (4)	41.8	30.7	53.5	318	843	4.03 (0.045)	90.6	<0.001	**4.38 (0.04)**
	>24.38 (4)	23.6	12.1	37.6	327	1470		97.0	<0.001	
Assessment tools (SA)^a^	Interview or and records (21)	27.0	21.4	33.0	2945	10 084	0.015 (0.904)	97.5	<0.001	0.002 (0.97)
	Others (7)	26.3	17.8	35.8	412	1672		94.2	<0.001	
Economic group	Low income (2)	10.2	7.9	12.8	68	584	177.96 (<0.001)	–	–	**53.29 (<0.001)**
	Lower middle income^a^ (2)	7.7	5.4	10.5	35	440		–	–	
	Upper middle income^a^ (7)	19.3	11.9	28.0	483	2606		96.4	<0.001	
	High income^a^ (17)	35.3	31.7	38.9	2771	8126		89.0	<0.001	
Region	Sub-Saharan Africa (4)	11.0	3.6	21.8	166	1208	71.47 (<0.001)	96.1	<0.001	**32.83 (<0.001)**
	East Asia and Pacific (7)	23.9	14.3	35.2	448	2288		97.1	<0.001	
	South Asia (1)	10.0	6.7	14.2	27	270		–	–	
	Europe and Central Asia (7)	32.2	27.4	37.2	1829	5463		89.3	<0.001	
	North America (8)	35.9	29.8	42.2	887	2527		90.0	<0.001	
Total (28)		26.8	22.1	31.9	3357	11 756	0.019	97.0	<0.001	−

a SA, suicide attempt. Bolded values: *p* < 0.05.

Meta-regression analyses revealed that sample size (slope  =  0.0001, *p*  =  0.91), mean age (slope = −0.006, *p*  =  0.42), the percentage of women (slope  =  0.0008, *p*  =  0.81), publication year (slope = −0.004, *p*  =  0.187) and assessment score (slope  =  0.04, *p*  =  0.07) did not statistically moderate the lifetime prevalence of suicide attempts.

### Sensitivity analysis and publication bias

**Fig. 3.**
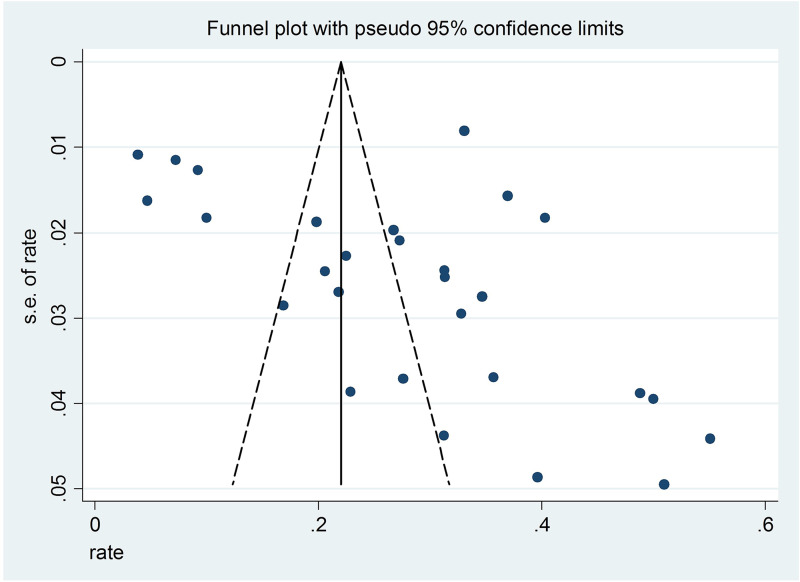
Funnel plot of the 28 included studies reporting the lifetime prevalence of suicide attempts.

Sensitivity analyses found that after removing each study sequentially, the results of the lifetime prevalence did not change significantly. The funnel plot showed slight asymmetry, but the Egger's tests (*t*  =  1.89, 95% CI −0.45 to 10.92, *p*  =  0.07) did not reveal any publication bias (Fig. 3).

## Discussion

This was the first meta-analysis that examined the prevalence of suicide attempts in individuals with schizophrenia across studies worldwide. This meta-analysis found that the lifetime prevalence of suicide attempts was 26.8% (95% CI 22.1–31.9%), which is approximately two times higher compared to the corresponding figure (14.6%, 95% CI 9.1–22.8%) in China (Dong *et al*., 2017). In addition, the prevalence in individuals with schizophrenia is much higher than the corresponding figure in general populations among 17 countries (2.7%) (Nock *et al*., 2008) and in China alone (0.8%, 95% CI 0.7–0.9%) (Cao *et al*., 2015). Apart from the confounding effects of study characteristics, clinical factors, such as severity of psychiatric symptoms, comorbid disorders and the stigma and discrimination related to the illness, could contribute to the higher risk of suicide attempts in individuals with schizophrenia (Hor and Taylor, 2010; Fuller-Thomson and Hollister, 2016; Duko and Ayano, 2018).

The pooled prevalence of suicide attempts from illness onset (45.9%) was highest, followed by the 6-month prevalence (38%) and the lifetime prevalence (26.8%). Prevalence estimates are significantly influenced by the illness severity and duration of the study. As in the case of this meta-analysis, only one study reported the 6-month prevalence of suicide attempt and two studies reported from-onset prevalence. This may bias the validity of the pooled prevalence of suicide attempts across studies with different timeframes and sampling. Other than the confounding effects caused by potential recall bias and small number of studies, various factors such as more severe psychotic symptoms, impaired global functioning from onset and stigma could increase the risk of suicidal behaviours in individuals with schizophrenia (Kaplan and Harrow, 1996; Radomsky *et al*., 1999; Assefa *et al*., 2012; Jakhar *et al*., 2017). Patients with a younger age of illness onset had a higher risk of suicide attempts, which is consistent with some (Panariello *et al*., 2010; Vinokur *et al*., 2014; Niehaus *et al*., 2004), but not all studies (Popovic *et al*., 2014). In contrast, individuals with schizophrenia with late-onset illness may have relatively better developed social skills and functioning, and less violent or impulsive tendency, all of which could reduce the risk of suicidality (Patterson *et al*., 1989; Vinokur *et al*., 2014).

Individuals with schizophrenia in high-income countries were more likely to attempt suicide than those in the low- or/and middle-incomes countries, while those in North America or Europe and Central Asia had a higher prevalence of suicide attempts than in South Asia, sub-Saharan Africa, East Asia and Pacific areas. The varying prevalence of suicide attempts across different regions could be partly explained by the differences in sociocultural and economic contexts and health care policies. For example, accessible mental health services and resources could effectively lower the risk of suicidal behaviours (Cooper *et al*., 2006), while societal discrimination of individuals with schizophrenia could lead to internalised stigma and increase the risk of suicide attempt (Assefa *et al*., 2012). In addition, religious and cultural factors are associated with the prevalence of substance abuse, such as alcohol and cocaine (Karch *et al*., 2006), which could in turn increase the risk of suicide attempt (Prince, 2018). Further, very few studies on suicide in schizophrenia have been conducted in low- and middle-income countries, which could lead to biased results. Of the included studies reporting lifetime prevalence, only two were conducted in low-income countries, two in lower middle income countries, one in South Asia and four in sub-Saharan Africa, which could reduce the reliability of the results. Apart from two studies in Turkey (upper middle-income country) (Uzun *et al*., 2009; Togay *et al*., 2015), studies in other countries were in the North America, Europe and Central Asia groups representing high-income countries. The relatively well-established reporting system for suicidal behaviours in these countries may be another reason for the higher prevalence of suicide attempts.

Gender difference in the prevalence of suicide attempts in individuals with schizophrenia has been controversial. For example, in some studies, females had more frequent suicide attempts (Tang *et al*., 2007; Fuller-Thomson and Hollister, 2016), while the opposite was found in other studies (Ran *et al*., 2015; Shrivastava *et al*., 2016). We did not find any gender difference, which is consistent with some (Dong *et al*., 2017), but not all studies (Hawton, 2000). Unlike the findings in previous studies (Hor and Taylor, 2010; Zhang *et al*., 2013), we did not find any association between younger age and risk of suicide attempts. Different illness phases and settings (e.g. inpatients *v*. outpatient settings) are associated with different risk of suicide for individuals with schizophrenia (Drake *et al*., 1985). However, subgroup analysis did not find any difference in suicide attempt prevalence between inpatients, outpatients and those in community.

Several methodological limitations need to be addressed. First, only studies published in English were included. Second, subgroup and meta-regression analyses were only performed for lifetime prevalence of suicide attempts due to a low number of studies of other timeframes. Third, some factors related to suicide attempts, such as prescription of antipsychotic medications, psychiatric comorbidities and number of admissions (Fuller-Thomson and Hollister, 2016), were not examined due to lack of data in the included studies. Fourth, similar to other meta-analyses (Winsper *et al*., 2013; Long *et al*., 2014; Li *et al*., 2016; Mata *et al*., 2015), high heterogeneity remained in the subgroup analyses, which is difficult to avoid in a meta-analysis of observational surveys. The heterogeneity was probably related to certain unmeasured factors, such as severity of psychotic symptoms, family history of psychiatric disorders and suicide, use of psychotropic medications and access to health services. In addition, only individuals with schizophrenia were included in this study, therefore the findings cannot be generalised to those with schizoaffective or schizophrenia spectrum disorders. Finally, only one study reported the 6-month prevalence of suicide attempt, two studies reported the 1-year prevalence, two studies reported the 1-month prevalence and two studies reported from-onset prevalence. Hence, the small number of studies in these timeframes may bias the validity of the pooled prevalence of suicide attempts.

In conclusion, suicide attempts are common in individuals with schizophrenia, especially those with an early age of onset and living in high-income countries and regions. Careful screening and effective preventive measures should be implemented routinely for this population.

## Supplementary material

For supplementary material accompanying this paper visit http://dx.doi.org/10.1017/S2045796019000313. click here to view supplementary material

## Availability of data and materials

All the data involved have been included in Tables and Figures of this manuscript.
