# Supplementary material for: Prevalence of suicide attempts in individuals with schizophrenia: a meta-analysis of observational studies
Source: Epidemiol Psychiatr Sci. 2019 Jun 7;29:e39. doi: 10.1017/S2045796019000313 (PMC8061230; doi:10.1017/S2045796019000313)
Supplement: Supplementary file 1 [file S2045796019000313sup001.docx]

Supplemental Table 1. Quality assessment of the included studies

| Assessment items | Yes, n (%) |
| --- | --- |
| 1. Are the study design and sampling method appropriate for the research question? | 35 (100) |
| 1. Is the sample representative of the target population? | 35 (100) |
| 1. Is the estimation method of sample size described in detail? | 0 (0) |
| 1. Are objective, suitable and standard criteria used for measurement of the health outcome? | 35 (100) |
| 1. Is the health outcome measured in an unbiased fashion? | 35 (100) |
| 1. Is the response rate adequate (>70%)? Are the refusers described? | 8 (22.9) |
| 1. Are the estimates of prevalence or incidence given with confidence intervals and in detail by subgroup, if appropriate? | 15 (42.9) |
| 1. Are the study subjects and the setting described in detail and similar to those of interest to you? | 35 (100) |
